# Structural Parasitology Meets Nanomedicine: Current Structural Evidence and Future Opportunities for *Leishmania* and *Schistosoma* Targets

**DOI:** 10.3390/cimb48101007

**Published:** 2026-09-30

**Authors:** Olasunkanmi Kayode Awote, Hammed Ayantola Onilude, Abidemi Paul Kappo

**Affiliations:** 1 Molecular Biophysics and Structural Biology Group, Department of Biochemistry, Faculty of Science, University of Johannesburg, Auckland Park, Johannesburg 2006, Gauteng, South Africa; oawote@uj.ac.za; 2 Drosophila Research, Drug Discovery, and Drug Delivery Group, Department of Biochemistry, Faculty of Science, Lagos State University, Ojo, Lagos PMB 0001, Lagos State, Nigeria; 3 Advanced Research Centre, School of Engineering, University of Glasgow, Glasgow G12 8QQ, UK; hammedonilude2@gmail.com

**Keywords:** cryo-EM, structural parasitology, nanomedicine, *Leishmania*, *Schistosoma*, membrane proteins, lipid nanoparticles

## Abstract

Neglected tropical diseases (NTDs) caused by *Leishmania* and *Schistosoma* species continue to impose a significant health burden in endemic regions, largely due to limited therapeutic options, drug resistance, and incomplete understanding of parasite biology at the molecular level. A major restriction in the development of targeted interventions is the structural inaccessibility of parasite membrane-associated proteins, which are central to host invasion, nutrient transport, immune evasion, and survival. Recent advances in cryogenic electron microscopy (cryo-EM) have expanded the structural biology toolkit available for studying difficult parasite proteins, particularly large and membrane-associated macromolecular assemblies. However, experimentally resolved cryo-EM structures remain limited for many of the *Leishmania* and *Schistosoma* targets relevant to therapeutic and vaccine development. This review therefore distinguishes experimentally determined structures from partial structural information and computational models and examines how potential cryo-EM approaches may address unresolved structural questions. We discuss current structural knowledge of selected parasite targets, approaches for recombinant expression and membrane-protein stabilization, membrane-mimetic systems for structural characterization, and computational methods that complement experimental structural biology. We further examine how experimentally supported structural information, complemented by computational predictions where appropriate, could contribute to structure-guided drug discovery, vaccine design, and targeted nanomedicine. At present, however, the direct translation of parasite structural information into rational nanotherapeutic design remains limited. Key gaps include the scarcity of experimentally resolved parasite membrane protein structures, challenges in their expression and structural characterization, and limited integration of structural biology with nanoparticle engineering. Resolving these gaps may improve the prospects for structure-informed therapeutic and nanomedicine applications against leishmaniasis, schistosomiasis, and other NTDs.

## 1. Introduction

Neglected tropical diseases remain among the most persistent infectious diseases worldwide, disproportionately affecting populations in regions with limited healthcare infrastructure and economic resources [1]. Leishmaniasis and schistosomiasis are among the major neglected tropical diseases (NTDs), affecting millions of people across Africa, Asia, Latin America, and parts of the Middle East [2]. Together, these parasitic infections contribute substantially to morbidity, disability, and socioeconomic burden, particularly in low- and middle-income countries where access to healthcare resources is often limited [3]. Substantial progress has been made in disease-control programmes; however, chemotherapy remains the principal treatment approach [4]. The effectiveness of existing drugs is constrained by toxicity, poor selectivity, prolonged treatment regimens, variable efficacy, and the emergence of drug-resistant parasite populations [5,6]. These limitations continue to drive the search for more selective therapeutic approaches that target parasite-specific biological processes, and understanding parasite biology is essential for developing such interventions.

Among the diverse molecular systems that sustain parasite survival and pathogenicity, membrane-associated proteins occupy a central position [7]. These proteins regulate nutrient acquisition, ion transport, environmental sensing, host–parasite communication, immune evasion, and drug uptake [8]. In *Leishmania*, membrane transporters and surface-associated proteins contribute to intracellular survival and adaptation within macrophages [9]. Similarly, *Schistosoma* species rely on tegumental proteins and membrane complexes to facilitate nutrient absorption, host interaction, and protection from immune attack [10]. Because many of these proteins are exposed on the parasite surface or positioned at critical biological interfaces, they represent attractive targets for therapeutic intervention, vaccine development, and targeted drug delivery [11]. However, the structural study of parasite membrane proteins has progressed more slowly than that of soluble proteins. Many of these membrane-associated proteins possess extensive hydrophobic domains, exhibit substantial conformational flexibility, and depend on complex lipid environments to maintain their native structure [12]. These characteristics often complicate protein expression, purification, and stabilization outside the native cellular membranes; thus, conventional structural techniques, particularly X-ray crystallography, have often struggled to capture physiologically relevant conformations [13]. Consequently, many parasite membrane proteins from *Leishmania* and *Schistosoma* remain structurally underexplored, limiting efforts to understand their molecular mechanisms and therapeutic potential.

Recent advances in cryogenic electron microscopy (cryo-EM) have improved the study of complex biological macromolecular assemblies that were previously difficult to characterize. Unlike traditional structural techniques that often require extensive crystallization or sample manipulation, cryo-EM enables visualization of proteins and molecular assemblies in a near-native, frozen-hydrated state [14]. The combined improvements in electron detectors, image processing, computational reconstruction, and sample preparation approaches have enhanced the resolution attainable by cryo-EM and broadened its applications to membrane proteins, large molecular complexes, and dynamic biological systems [15]. These developments have improved previously difficult-to-characterize structures and have also provided new insights into parasite biology at the molecular level, extending the results generated by cryo-EM beyond protein characterization alone. The high-resolution structural models from cryo-EM can expose receptor-binding regions, structurally exposed domains, conformational states, and ligand-interaction sites that may be exploited during therapeutic design [16]. Such information is also becoming increasingly relevant to nanomedicine, where targeted drug delivery systems depend largely on precise interactions between nanocarriers and biological targets [17]. This structural information can also inform the design of nanoparticles that improve cellular uptake, enhance drug accumulation, and mitigate unintended interactions with host tissues [18].

Cryo-EM studies involving infectious-disease protein targets are now an important tool in structural biology, but their application in antiparasitic nanomedicine remains limited. Many studies have examined structural determinants of infection and nanoparticle development as separate areas, with relatively few investigating how experimentally resolved parasite structures could inform targeted nanoparticle design once target accessibility, binding specificity, and delivery efficacy have been experimentally established. As a result, a gap persists between advances in structural parasitology and translational nanomedicine for neglected tropical diseases. Previous reviews have largely examined parasite structural biology, cryo-EM methodology, or nanomedicine as separate areas, including reviews focused on nanotechnology-based treatment of leishmaniasis and schistosomiasis [19,20]. The contribution of the present review is not to propose a new validated nanomedicine platform, but to critically examine the extent to which currently available structural evidence for selected *Leishmania* and *Schistosoma* targets can support therapeutic and nanomedicine-related applications. This narrative, mechanistic review focuses on selected *Leishmania* and *Schistosoma* targets for which experimentally determined structures, partial structural information, or computational models are available, and examines how these structural data relate to drug discovery, vaccine design, and targeted nanomedicine. The review therefore asks three related questions: which parasite targets currently have experimentally supported structural information; which unresolved targets and structural features could be investigated using cryo-EM, cryo-electron tomography (cryo-ET), or complementary computational approaches; and to what extent can these structural data support drug discovery, vaccine design, and targeted nanomedicine? Particular attention is given to the distinction among experimentally determined structures, partial structural information, and computational models, and to the biological requirements that must link structural features to target accessibility, ligand recognition, nanocarrier design, and antiparasitic activity. By comparing these elements across the two parasite genera, this review identifies where structure-guided drug discovery, vaccine design, and targeted delivery are supported by existing evidence and where proposed applications remain prospective. This distinction is important because the availability of a structural model alone does not establish that a parasite target is accessible, selectively bindable, or suitable for nanoparticle-mediated delivery. In addition, we identify where the available evidence supports structure-guided therapeutic applications and where further experimental validation is required before structural information can be translated into targeted nanomedicine. Table 1 shows the major challenges in leishmaniasis and schistosomiasis, consequences for disease management, and the potential future contribution of cryo-EM.

The cryo-EM applications listed in Table 1 describe potential future uses for the structural investigation of these therapeutic problems and should not be interpreted as established cryo-EM outcomes for *Leishmania* or *Schistosoma* targets. Despite these significant advances in structural biology, experimentally resolved membrane protein structures from *Leishmania* and *Schistosoma* remain relatively limited compared with those available for bacterial, viral, and mammalian systems. This structural gap reflects persistent challenges in membrane protein expression, purification, stabilization, and preservation of native structural states during experimental analysis [35]. It also limits the extent to which experimentally resolved structures can currently support structure-guided therapeutic development for these parasites. Therefore, understanding the potential contribution of cryo-EM remains important for antiparasitic drug discovery and nanomedicine [36]. To distinguish established structural evidence from computational inference, the targets discussed in this review are classified according to the highest level of structural information currently available. Accordingly, structural evidence is reported according to its experimental status throughout the review: experimentally determined structures are distinguished from experimentally characterized domains or regions, homologous structures, and computational predictions. Docking and molecular dynamics results are treated as hypothesis-generating analyses rather than direct structural evidence. Experimentally resolved cryo-EM or cryo-electron tomography (cryo-ET) structures are distinguished from structures obtained using other experimental approaches, including X-ray crystallography and nuclear magnetic resonance (NMR). Partial structural information, such as experimentally characterized domains or protein regions, is considered separately from complete protein structures, whereas AlphaFold predictions, homology models, docking studies, and other computational approaches are treated as structural models rather than experimentally determined structures. Functional characterization of a target is likewise distinguished from structural determination, and docking, molecular dynamics, and other computational analyses are treated as hypothesis-generating approaches rather than as experimental structural evidence. This classification is particularly important for *Leishmania* and *Schistosoma*, where experimentally characterized targets such as Glycoprotein 63 (GP63) and *Schistosoma mansoni* tetraspanin-2 (Sm-TSP-2) have structural information available through X-ray crystallography and NMR, respectively, but these structures do not represent complete cryo-EM structures of the corresponding native membrane-associated proteins [28,29].

### Literature and Structural Database Search

The literature relevant to the structural biology, target characterization, cryo-EM/cryo-ET applications, computational structural analysis, and nanomedicine of *Leishmania* and *Schistosoma* was identified through searches of PubMed/MEDLINE, Scopus, and Web of Science using combinations of parasite names, target names, structural methods, and nanomedicine-related terms. Structural records were additionally examined in the Protein Data Bank (PDB) and Electron Microscopy Data Bank (EMDB) to identify experimentally determined atomic structures, electron microscopy density maps, and associated structural information. The PDB provides experimentally determined three-dimensional macromolecular structures, whereas EMDB archives electron microscopy density maps and tomographic volumes; corresponding atomic models derived from electron microscopy data may be deposited in the PDB. Searches and structural-record checks were conducted up to May 2026. Retrieved studies were screened for relevance to the parasite targets and the structural or therapeutic questions addressed in this review, with primary experimental studies prioritized for target-specific evidence, and review articles used primarily for broader biological and methodological context.

## 2. Structural Parasitology in the Cryo-EM Era

The application of structural biology to parasitic diseases has historically lagged behind that of bacterial, viral, and mammalian systems. Many parasite proteins exhibit biochemical characteristics that complicate experimental studies, including conformational flexibility and extensive post-translational modification [37]. These challenges have limited the availability of high-resolution structures for numerous molecular targets involved in parasite survival, host adaptation, and therapeutic resistance. Notwithstanding, the increasing availability of structural information has supported efforts to understand parasite protein functions and to exploit them as molecular therapeutic targets. In addition, translational applications such as vaccine development, ligand-directed drug delivery, and nanoparticle design are starting to benefit from these developments [38]. Accordingly, cryo-EM is increasingly being incorporated as a tool for structural characterization and may provide information that can subsequently support therapeutic design when combined with target validation and functional studies [39].

### 2.1. Why Parasite Membrane Proteins Remain Difficult to Study?

Parasite membrane proteins present target-specific structural difficulties because their hydrophobic transmembrane regions, conformational flexibility, and dependence on membrane lipids complicate expression, purification, and structural stabilization. Many of these proteins are located on the parasite surface or directly involved in host interactions, making them attractive therapeutic targets [40]. However, their hydrophobic transmembrane regions and dependence on membrane lipids can make removal from biological membranes unstable, leading to aggregation, loss of activity, or conformational rearrangement [41]. These properties often limit the production of homogeneous samples suitable for structural analysis.

In *Leishmania*, membrane-associated proteins such as aquaglyceroporins, nutrient transporters, ion channels, and surface metalloproteases contribute to parasite survival, metabolic adaptation, and virulence within hostile host macrophages [42]. Parasite-specific features further complicate structural characterization. Many surface-associated proteins in *Leishmania* are embedded within a highly specialized membrane environment and may be influenced by the surrounding glycocalyx and lipophosphoglycan (LPG)-rich surface layer. These components can affect protein accessibility, intermolecular interactions, and the physical environment encountered during purification and structural analysis [43]. Similarly, *Schistosoma* species possess specialized tegument-associated proteins and complex membrane transport systems that mediate essential nutrient acquisition, host immune modulation, and physical host–parasite interactions [44]. High-resolution characterization of these systems could help define molecular interfaces, binding sites, and conformational features relevant to therapeutic intervention; however, the extent to which such structural information translates into drug or vaccine development remains target-dependent and requires experimental validation. Another challenge arises from the strong influence of lipid–protein interactions on membrane protein behaviour [45]. Lipids do not merely provide structural support for biological membranes; they often participate directly in protein stabilization, conformational regulation, and signalling processes. Removing these native lipid matrices during standard extraction procedures can disrupt critical allosteric interactions, thereby collapsing the target protein into an inactive conformation [45]. Hence, structural studies of membrane proteins may require membrane-mimetic environments that better preserve native-like lipid–protein interactions and conformational states.

The tegument in *Schistosoma* forms a specialized syncytial surface that contains a complex array of proteins and membrane-associated components involved in host–parasite interactions, nutrient acquisition, and immune modulation [44]. Consequently, an isolated recombinant protein may not fully recapitulate the structural organization or accessibility of its counterpart in the native tegument. Membrane-associated parasite proteins present target-specific structural difficulties rather than a single general limitation. Aquaporin-1 (AQP1) and Schistosome Glucose Transporter Protein 4 (SGTP4) are small membrane proteins for which limited extramembrane mass can reduce particle contrast and complicate single-particle cryo-EM analysis. ATP-binding cassette (ABC) transporters are larger but may require preservation of membrane-dependent conformations and lipid interactions. Glycoprotein 63 (GP63) and *Schistosoma mansoni* tetraspanin-2 (Sm-TSP-2) introduce additional considerations because their extracellular regions, membrane association, glycosylation, and native surface environments may influence structural organization and accessibility. In *Schistosoma*, the specialized tegument further complicates extrapolation from isolated recombinant proteins to their native parasite context. These differences support target-specific expression, membrane stabilization, and structural approaches rather than treating all parasite membrane proteins as equivalent experimental systems [46,47,48,49,50]. The choice of expression system, purification conditions, and membrane-mimetic environment must therefore match the biological and structural characteristics of each target, with detergent screening, construct optimization, chromatographic purification, and stabilization in lipid- or polymer-based systems providing practical steps to improve sample quality before cryo-EM analysis. Computational predictions can provide useful hypotheses regarding topology, conformational states, and potential binding sites, but these models should not be considered equivalent to experimentally resolved structures.

Table 2 provides a comparative summary of the principal parasite targets discussed in this review, including their biological location, function, therapeutic relevance, and current structural status. The comparison also indicates whether available structural information derives from a complete experimentally determined structure, a characterized domain or region, homologous structures, or computational modelling. This organization emphasizes that biological importance and structural characterization do not necessarily occur at the same level across the selected targets. For AQP1, SGTP4, and several parasite-specific ABC transporters, functional or computational evidence exists, but no corresponding experimentally resolved full-length structure is available. GP63 and Sm-TSP-2 provide a different situation: experimental structural information is available, but it represents solubilized GP63 and the isolated EC2 domain of Sm-TSP-2 rather than the intact membrane proteins in their native parasite environments [28,29,49]. These examples, therefore, should not be treated as equivalent levels of structural evidence. Rather, they illustrate a continuum ranging from functional characterization and computational prediction to experimentally determined partial or solubilized structures. This distinction is important when considering whether structural information can support mechanistic interpretation, target validation, or future structure-informed therapeutic development.

### 2.2. Current Therapies and Structural Evidence for Major Parasite Targets

Current chemotherapy remains the primary treatment strategy for both leishmaniasis and schistosomiasis, yet many available drugs are limited by toxicity, prolonged treatment courses, emerging resistance, or incomplete efficacy across parasite species and life stages [25,26]. These limitations reinforce the need to identify structurally characterized parasite targets that can support more selective therapeutic development. For leishmaniasis, the principal therapies include liposomal amphotericin B, miltefosine, paromomycin, and pentavalent antimonials, although treatment recommendations vary according to parasite species and geographic region. Liposomal amphotericin B remains a cornerstone of visceral leishmaniasis treatment in many settings [59], whereas miltefosine represents the first orally available anti-leishmanial agent and has broadened treatment options despite concerns regarding resistance and species-dependent efficacy [60]. Pentavalent antimonials continue to be used in several endemic regions, although their clinical utility has been reduced by toxicity and declining effectiveness in areas with established resistance [22].

From a structural perspective, however, many clinically relevant *Leishmania* targets remain incompletely characterized. GP63 is one of the representative *Leishmania* targets for which partial experimentally determined three-dimensional structural information is available [28], whereas AQP1 and several parasite-specific ABC transporters continue to rely largely on functional studies and computational modelling. This disparity illustrates how current therapy frequently precedes detailed structural understanding and highlights the opportunity for cryo-EM to clarify unresolved membrane-associated targets that may contribute to future structure-guided drug discovery. For schistosomiasis, praziquantel remains the WHO-recommended first-line treatment and continues to underpin mass drug administration programmes because of its broad effectiveness against adult worms. Nevertheless, praziquantel exhibits reduced activity against immature parasites, and concerns about reliance on a single frontline therapy have spurred interest in identifying additional therapeutic targets and complementary treatment methods [23,26].

Structural evidence for *Schistosoma* similarly remains uneven. Although the EC2 domain of Sm-TSP-2 has been experimentally characterized, many tegument-associated proteins, transporters, and membrane complexes that represent attractive therapeutic targets remain structurally unresolved [29,49]. Consequently, current chemotherapy and available structural knowledge remain imperfectly aligned across both parasite systems, underscoring the importance of cryo-EM and complementary structural approaches to expand the repertoire of experimentally validated targets and support future drug design, vaccine, and nanomedicine development [24,27,35,61].

### 2.3. Experimentally Determined Parasite Structures and Their Relevance to Therapeutic Development

Experimentally determined structures provide different levels of information depending on whether they represent an intact protein, an isolated domain, a solubilized construct, or a structure obtained from a homologous system. This distinction matters for *Leishmania* and *Schistosoma*, where many therapeutically relevant membrane proteins remain structurally unresolved. X-ray crystallography and solution NMR have nevertheless provided useful structural information for selected parasite proteins or domains [28,29]. Rather than treating these structures as direct substitutes for intact membrane-protein structures, they provide experimentally supported reference points from which structural hypotheses can be developed and tested. The evidence discussed for the principal parasite targets was therefore prioritized toward primary studies reporting experimental structures, target-associated functional or ligand-binding data, target validation, and antiparasitic delivery, with review articles used mainly to provide broader biological or methodological context. For the purposes of this review, it is important to distinguish three levels of evidence: experimentally established structural information, technically plausible structural opportunities, and prospective therapeutic applications. The first is supported by available structural data; the second concerns approaches that could reasonably be applied to currently unresolved parasite targets; and the third requires additional validation of target accessibility, specificity, formulation, and biological efficacy.

**Figure 1:**
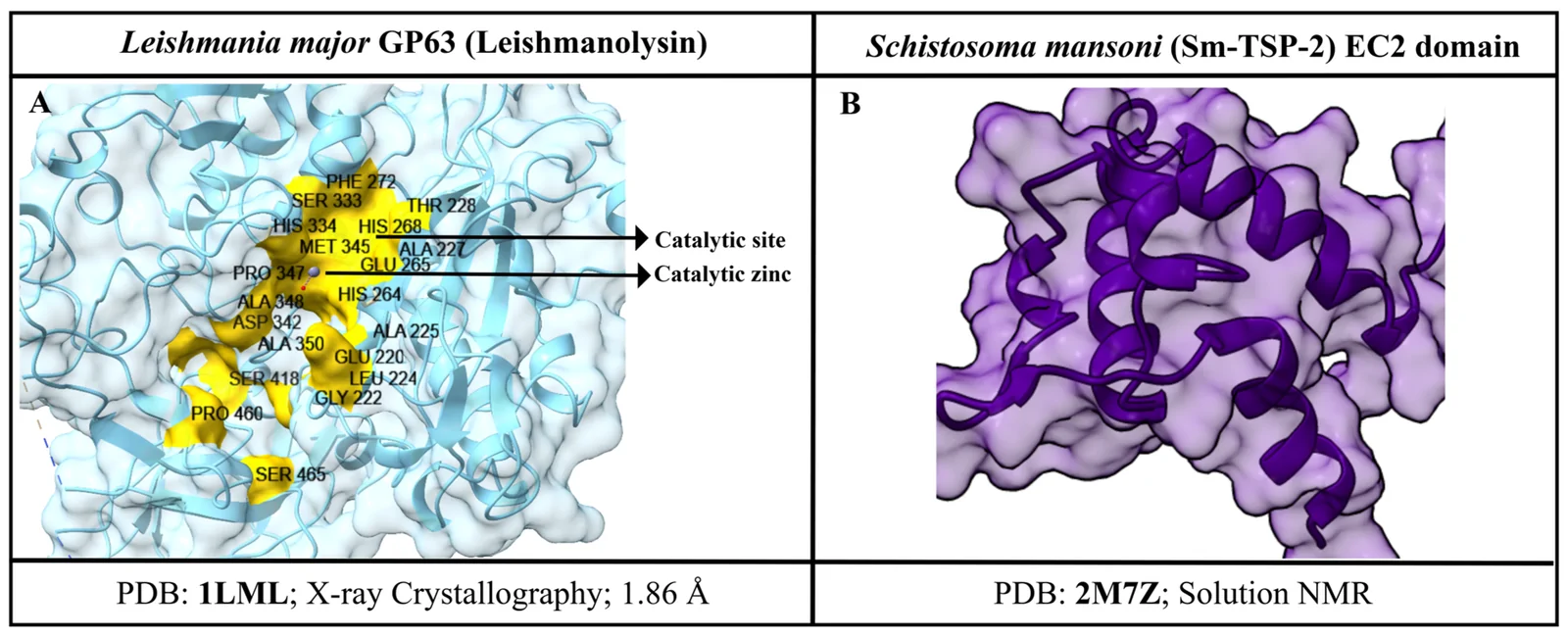
Molecular representation of experimentally characterized structural regions of selected *Leishmania* and *Schistosoma* targets (extracted from the PDB database and visualized with ChimeraX software). (**A**) X-ray crystal structure of *Leishmania major* leishmanolysin (GP63; PDB 1LML), showing the experimentally determined protein architecture and catalytic zinc-containing region. (**B**) Solution NMR structure of the extracellular EC2 domain of *Schistosoma mansoni* Sm-TSP-2 (PDB 2M7Z). The Sm-TSP-2 structure represents an 81-residue extracellular domain and does not resolve the complete four-pass membrane protein or its organization within the native tegument. The structures illustrate the different levels and experimental methods of structural evidence available for these parasite targets.

For Leishmania, GP63 provides a representative example of how experimental structural information can complement functional studies. The available X-ray structure of solubilized *L. major* leishmanolysin provides experimentally supported information on its three-dimensional organization and catalytic architecture [28] but does not resolve the intact membrane-associated protein in its native parasite environment. Such information can provide a structural basis for investigating inhibitor binding and antigenic regions [28,62,63]. For *Schistosoma mansoni*, solution NMR has provided structural information for the extracellular EC2 domain of Sm-TSP-2 (PDB 2M7Z), an 81-residue fragment of the full-length tetraspanin [29]. This provides experimentally supported domain-level structural information, but not the structure of the intact four-pass membrane protein. Figure 1 provides molecular representations of the two experimentally characterized parasite targets (GP63 and Sm-TSP-2) and illustrates the difference between a structurally characterized protein domain and an experimentally determined structure of a larger parasite surface protein [28,29].

**Figure 2:**
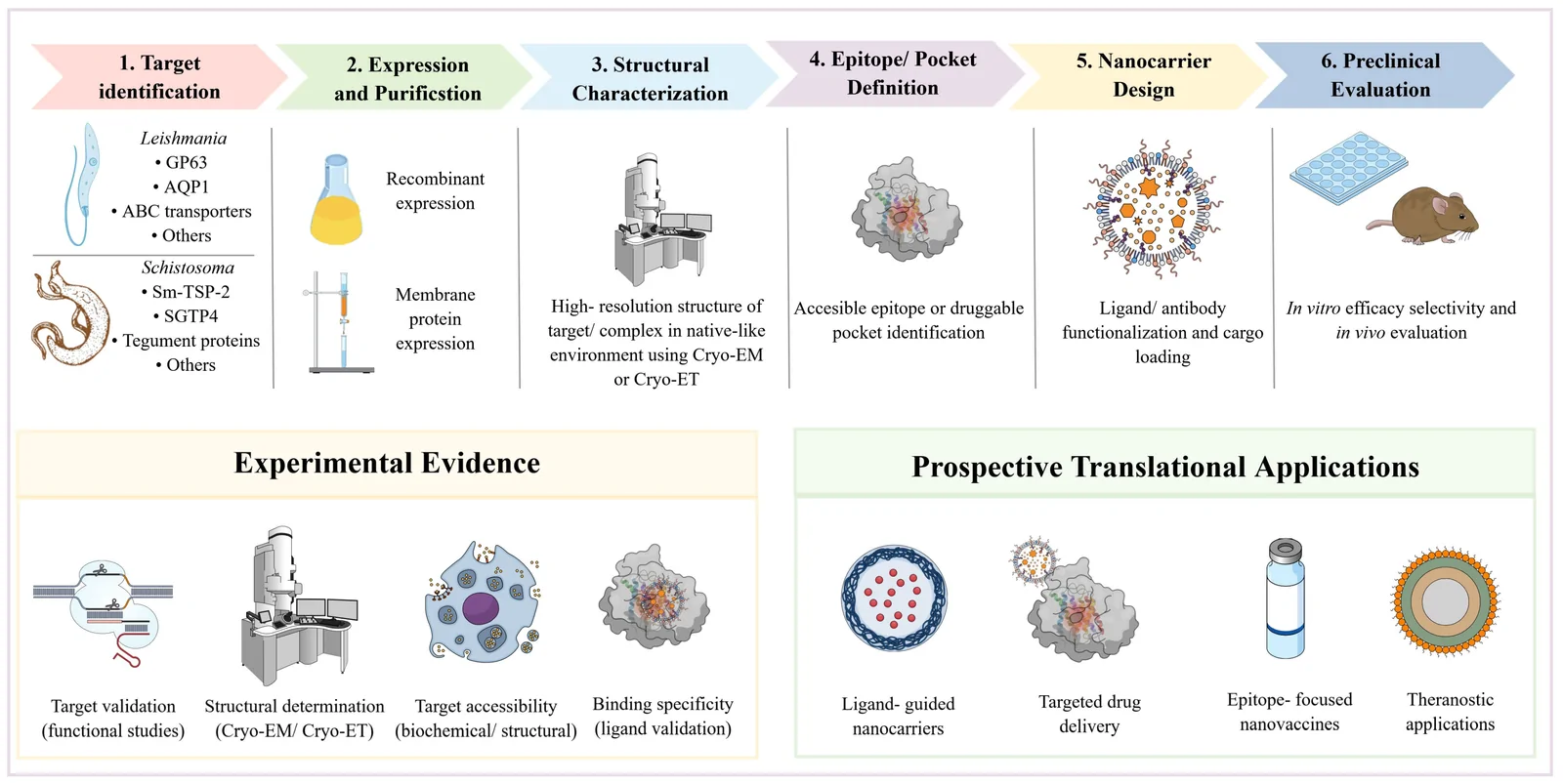
From Parasite-Targeted Identification to Structure-Guided Nanotherapeutic Development (created with Mind the Graph software: https://mindthegraph.com). Conceptual workflow linking parasite target identification and structural evidence assessment with cryo-EM/cryo-ET characterization, computational analysis, identification of candidate binding pockets or accessible epitopes, nanocarrier functionalization, and subsequent *in vitro* and *in vivo* validation. Experimentally resolved structures represent the strongest structural basis for this workflow, whereas computationally predicted structures provide hypothesis-generating information that requires experimental validation.

Cryo-EM has expanded the structural analysis of membrane proteins and large macromolecular assemblies, including systems that are difficult to maintain in forms suitable for conventional crystallography [24,27]. For the parasite targets considered here, however, their value should be assessed on a target-by-target basis rather than assumed from successes in other biological systems. A cryo-EM structure of a homologous transporter, receptor, or membrane complex can establish methodological precedent, but it does not constitute structural evidence for the corresponding *Leishmania* or *Schistosoma* protein. The same distinction applies to ligand-directed nanomedicine: targeting approaches validated in non-parasitic systems may provide methodological precedent for nanocarrier functionalization or ligand selection, but they should not be interpreted as evidence of parasite-specific ligand recognition unless binding and biological activity have been demonstrated using the relevant *Leishmania* or *Schistosoma* target. Conversely, an experimentally resolved parasite domain or solubilized protein can provide useful structural constraints without representing the complete native assembly. The most informative future studies will therefore need to address not only whether a target can be structurally resolved, but also whether the resulting preparation retains the membrane environment, oligomeric state, post-translational modifications, and protein interactions relevant to its biological function. From a nanomedicine perspective, experimentally determined structures can provide information that may eventually support the selection of accessible epitopes, ligand-binding sites, protein–protein interfaces, or other targetable structural features [19,63,64]. Nevertheless, structural determination alone does not establish that a site is accessible to a nanocarrier in the parasite, that ligand attachment will preserve binding activity, or that a structure-guided nanotherapeutic will demonstrate efficacy *in vivo*. Experimental target validation, accessibility studies, ligand optimization, nanocarrier formulation, and *in vitro* and *in vivo* evaluation remain necessary steps between structural characterization and therapeutic application. Figure 2 summarizes a proposed workflow in which parasite target identification and structural characterization can inform target-site definition and computational analysis, followed by nanocarrier design and experimental validation.

### 2.4. Structural Evidence for Leishmania Targets

Membrane transporters are among the most extensively investigated protein classes in *Leishmania* due to their direct involvement in nutrient acquisition and drug transport across biological membranes. These transport proteins regulate the uptake of essential metabolites and tightly influence cellular susceptibility to anti-leishmanial drugs. Therefore, alterations in transporter expression or functional mutations have been associated with clinical treatment failure and the spread of drug resistance. High-resolution characterization of these proteins could help clarify their transport mechanisms and identify molecular features that may subsequently be evaluated as therapeutic targets.

Aquaglyceroporin 1 (AQP1) is a specialized transmembrane channel that facilitates the passive transport of water, glycerol, and small uncharged solutes across biological membranes [46,51]. In *Leishmania* parasites, AQP1 has also been implicated in the uptake of trivalent antimonials, and reduced expression, gene deletion, or disruptive mutations are associated with reduced drug accumulation and decreased antimonial sensitivity [21,51,52]. Despite this functional evidence, a fully resolved experimental structure of *Leishmania* AQP1 remains unavailable. Its relatively small membrane-protein architecture presents an additional challenge for single-particle cryo-EM because the transmembrane region provides limited mass and surface features for reliable particle alignment, particularly when the protein is surrounded by detergent or lipid molecules. This problem could be further complicated by conformational or orientation heterogeneity during grid preparation. One possible experimental strategy would be to increase the effective particle mass and provide additional alignment features by forming AQP1 complexes with high-affinity fiducial binders such as Fab fragments, nanobodies, or larger engineered binders [65,66,67]. Such approaches have been used more broadly to facilitate structural analysis of challenging small membrane proteins, although their suitability for *Leishmania* AQP1 would need to be established experimentally. The use of a lipid nanodisc or another membrane-mimetic system could additionally provide a more physiologically relevant environment while preserving the transmembrane architecture. A full-length structure could clarify the organization of the channel, the structural basis of substrate and antimonial passage, and the effects of resistance-associated mutations. Such information could subsequently generate testable hypotheses for inhibitor development or targeted delivery, but these applications remain prospective until the relevant structural determinants and target accessibility are experimentally established.

Another important class of therapeutic targets comprises surface-associated proteins, particularly GP63 (leishmanolysin), a zinc-dependent metalloprotease abundantly expressed on the extracellular surface of *Leishmania* promastigotes [9]. GP63 contributes to host–cell interaction, immune modulation, and parasite survival [47,53]. An experimentally determined structure of *L. major* GP63 is available from X-ray crystallography (PDB 1LML), providing high-resolution information on the mature protease and its catalytic architecture [28]. However, this structure should not be interpreted as a structural representation of the intact membrane-associated protein in its native parasite environment. Native GP63 is GPI-anchored at the parasite surface and is extensively glycosylated, features that may influence its orientation, conformational behaviour, and accessibility to antibodies or other binding molecules. In addition, the dense glycocalyx and lipophosphoglycan (LPG)-rich surface of *Leishmania* may impose steric and biochemical constraints that are not reproduced by an isolated recombinant catalytic domain. These differences are important because a structurally accessible region in an isolated protein may not necessarily remain equally accessible on the parasite surface [43]. Structural studies using appropriately membrane-associated or membrane-mimetic GP63 preparations could therefore provide complementary information on the organization and accessibility of the native protein, although the feasibility of such approaches will depend on maintaining protein integrity and conformational homogeneity. Moreover, this may serve as a basis for generating testable hypotheses regarding ligand binding and epitope accessibility, but further biochemical, cellular, and *in vivo* studies will be required before such information can be translated into validated targeted nanotherapeutic platforms.

The ATP-binding cassette (ABC) transporters are another class of membrane-embedded proteins that mediate active transport and have been implicated in multidrug resistance (MDR). Up-regulated expression or increased catalytic activity of these efflux pumps expels chemically diverse therapeutic agents from the parasite cytoplasm [55]. This accelerated compound efflux reduces intracellular drug concentrations below effective thresholds, severely limiting the therapeutic efficacy of frontline anti-leishmanial regimens [48]. Nevertheless, high-resolution structural characterization of ABC transporters remains incomplete across most *Leishmania* species. Structural characterization of these multi-domain assemblies, particularly the relationship between their nucleotide-binding domains (NBDs) and transmembrane domains (TMDs), could help determine whether conserved or parasite-specific features are suitable for pharmacological modulation. Such structural information could eventually inform strategies for efflux modulation or delivery system design that improve intracellular drug exposure; however, these applications remain prospective in *Leishmania* because experimentally resolved structures of the relevant parasite ABC transporters are limited.

### 2.5. Structural Evidence for Schistosoma Targets

The structural characterization of *Schistosoma* species requires experimental approaches that differ from those used for protozoan parasites. Adult schistosomes survive for prolonged periods within the host bloodstream, enduring constant exposure to the host immune system, physical shear forces, and fluctuations in nutrient availability [10,34]. This tegument is central to the parasite’s survival; therefore, tegument-associated proteins are important candidate targets for drug development, vaccines, and targeted therapies [57,58]. As a result, many tegumental proteins remain poorly characterized structurally. The membrane association, extensive post-translational modification, conformational flexibility, dependence on specific lipid environments, and interactions with neighbouring tegumental components can complicate recombinant expression, purification, and structural homogeneity [10]. These factors also create an important interpretive limitation: a recombinant protein or isolated domain may adopt a structurally valid conformation yet fail to reproduce the orientation, oligomeric state, or accessibility of the corresponding protein in the native tegument. Recent advances in cryo-ET could provide complementary information on native tegumental organization, but successful application to intact *Schistosoma* membrane complexes remains an experimental question [68].

*Schistosoma mansoni* tetraspanin-2 (Sm-TSP-2), a tegumental membrane protein and vaccine candidate, provides a particularly clear example of the difference between domain-level structural information and the structure of an intact parasite membrane protein [49]. Experimental structural information is available for its surface-exposed EC2 domain, determined by solution NMR (PDB 2M7Z) [29,49]. The deposited structure contains the isolated 81-residue EC2 domain rather than the complete four-pass membrane protein [29]. Consequently, it provides valuable information about the local architecture of an exposed extracellular region but cannot by itself reveal how the EC2 domain is positioned relative to the transmembrane helices, the membrane, or other components of the tegumental environment. The distinction is structurally important because the EC2 domain is presented within a membrane-embedded four-pass protein and a densely organized tegumental environment in the parasite. Membrane topology, interactions with other tegumental proteins, lipid composition, and post-translational modifications could influence the conformation and accessibility of surface-exposed regions in the intact protein [29]. A future cryo-EM study could therefore focus on the full-length protein reconstituted in a suitable membrane environment, whereas cryo-ET may become more informative if the objective is to examine Sm-TSP-2 within native tegumental assemblies or its interactions with neighbouring proteins [24,27,68]. Such approaches could help determine whether the conformation and accessibility of the EC2 region are preserved in the intact membrane context. Until this has been established experimentally, the available EC2 structure should be regarded as a structural foundation for hypothesis generation rather than as a complete representation of the native Sm-TSP-2 architecture.

Glucose transporters represent another important class of *Schistosoma* targets because adult schistosomes depend heavily on glucose uptake to support their metabolism, growth, reproduction, and continuous tegumental maintenance. *Schistosoma* glucose transporter protein 4 (SGTP4), located predominantly at the apical tegumental membrane, has been implicated in glucose uptake from the host bloodstream [50,56]. As a membrane transporter embedded within the tegument, it is likely that its structural state depends on the surrounding lipid environment and the conformational cycle associated with substrate transport. Consequently, a recombinant SGTP4 preparation that does not reproduce the relevant membrane environment may not fully capture the conformational states or extracellular surfaces present in the parasite. Functional inhibition of glucose transport can impair parasite viability, supporting the therapeutic relevance of this transporter class [57]. However, unlike Sm-TSP-2, SGTP4 currently lacks an experimentally resolved high-resolution structure that could define its complete transmembrane architecture, substrate-binding pathway, and conformational states [50,55,56]. This presents a different structural challenge from that of an isolated extracellular domain, where the principal information required is distributed across the membrane-spanning helices and their associated extracellular and intracellular regions. A successful structural study would therefore need to preserve the transporter in a stable, membrane-compatible state and, ideally, capture multiple relevant conformational states. Detergent screening, lipid nanodiscs, or other membrane mimetics could help optimize construct stability and membrane compatibility before cryo-EM analysis [24,27]. If SGTP4 is strongly associated with other tegumental components in its native setting, cryo-ET could provide complementary information on its spatial organization that would not be available from an isolated recombinant transporter. A structure could subsequently inform the identification of substrate-binding or regulatory pockets and generate hypotheses for selective inhibition. However, any application of surface-targeted nanocarriers would remain prospective until the accessibility and biological relevance of candidate sites are experimentally established.

### 2.6. Comparative Structural Prospects in Leishmania and Schistosoma

*Leishmania* and *Schistosoma* species differ fundamentally in taxonomy, life cycles, and clinical pathogenesis, and both parasites depend on specialized surface and membrane-associated proteins for survival, metabolic adaptation, and interaction with the host. In *Leishmania*, surface metalloproteases (such as GP63) and specific nutrient transporters play major roles in facilitating intracellular persistence in macrophages and modulating therapeutic responses. Conversely, in *Schistosoma*, multi-subunit tegument-associated proteins and apical membrane transport complexes directly mediate host–parasite interactions and regulate vital metabolic flux from the host bloodstream [1]. Despite their divergent evolutionary paths, the dependence of both parasites on specialized surface and membrane-associated systems makes these proteins and complexes important candidates for comparative structural investigation and, potentially, subsequent structure-guided intervention.

Many surface proteins in both parasites remain structurally unresolved, but the constraints in their native contexts differ. In *Leishmania*, surface glycoconjugates and the LPG-rich glycocalyx can affect target accessibility, whereas in *Schistosoma*, the syncytial tegument and associated membrane-protein network complicate native-state characterization [43,44]. Single-particle cryo-EM and cryo-ET, therefore, provide complementary approaches, but their value will depend on the properties of each target and the extent to which native membrane organization can be preserved.

## 3. Experimental Systems for Cryo-EM Investigation of Parasite Targets

The application of cryo-EM to *Leishmania* and *Schistosoma* membrane proteins depends not only on advances in imaging technology but also on the ability to produce stable, structurally relevant protein samples. The targets discussed in this review differ substantially in their experimental requirements. AQP1 and SGTP4 are multi-pass transport proteins whose structural analysis depends on correct membrane insertion and conformational stability, whereas GP63 presents additional challenges related to its surface anchoring, glycosylation, and extracellular environment. Sm-TSP-2 similarly requires preservation of its membrane topology and extracellular architecture beyond the isolated EC2 domain [49]. Thus, expression-system selection should be guided by the biological and structural properties of each target rather than by protein yield alone. Bacterial, yeast, insect-cell, mammalian, and cell-free systems, therefore, represent complementary options for generating material suitable for biochemical characterization, membrane reconstitution, and, where feasible, cryo-EM analysis (Table 3).

### 3.1. Bacterial Expression Systems

Bacterial expression systems, particularly *Escherichia coli*, remain attractive because of their rapid growth, low cost, genetic tractability, and high protein yields [69,70]. However, their value for the parasite membrane proteins considered here is mainly supportive rather than definitive. The absence of parasite- or mammalian-like post-translational processing and the difficulty of maintaining correctly folded multi-pass membrane proteins can limit their suitability for intact targets [70]. These limitations are particularly relevant to AQP1, SGTP4, and parasite ABC transporters, whose structures depend on correct membrane insertion and lipid interactions. For the targets considered in this review, *E. coli* is therefore more appropriate for producing soluble domains, catalytic fragments, selected mutants, or constructs for biochemical and antibody-based studies than for routine production of intact GP63, AQP1, Sm-TSP-2, or SGTP4. Such preliminary constructs can nevertheless be valuable for mapping functional residues, testing ligand interactions, and optimizing constructs before moving to eukaryotic expression platforms.

### 3.2. Yeast Expression Systems

Yeast expression systems provide an important intermediate platform between bacterial and higher eukaryotic hosts for producing recombinant proteins. Among the available yeast species, *Komagataella phaffii* (formerly *Pichia pastoris*) has become one of the most widely used because it combines rapid growth, high cell-density cultivation, efficient secretion of recombinant proteins, and the ability to perform many eukaryotic post-translational modifications [71]. These characteristics have established *K. phaffii* as a versatile host to produce enzymes, vaccine antigens, antibodies, and membrane-associated proteins for structural and functional investigations. Compared with bacterial hosts, yeast expression systems generally produce proteins with improved folding and greater structural stability because they possess endoplasmic reticulum- and Golgi-mediated processing pathways. They also support disulfide bond formation and selected glycosylation events that are essential for the biological activity of many eukaryotic proteins. Although yeast glycosylation patterns differ from those of mammalian cells, advances in strain engineering and glycoengineering have substantially improved their suitability for recombinant protein production intended for structural biology and biopharmaceutical applications. *K. phaffii* offers several practical advantages, including higher quantities of recombinant membrane proteins than many mammalian platforms while maintaining relatively low production costs and scalable fermentation processes. In addition, its compatibility with fluorescence-based expression screening, affinity purification, and membrane protein stabilization workflows has increased its utility in cryo-EM studies [72]. For the parasite targets considered here, *K. phaffii* may be particularly useful when eukaryotic folding and disulfide bond formation are required, but exact mammalian glycosylation is not essential. This makes yeast a possible intermediate platform for selected soluble or membrane-associated regions of GP63 and Sm-TSP-2, as well as for preliminary production of parasite transport proteins. Its suitability for intact AQP1, SGTP4, or other highly membrane-dependent targets would, however, need to be established experimentally because yeast-derived membrane composition and glycosylation may influence folding, oligomerization, and conformational stability [71,72].

### 3.3. Baculovirus–Insect Cell Expression Systems

Baculovirus-mediated expression in insect cells remains one of the most widely used approaches for producing complex eukaryotic membrane proteins for structural studies [73]. This expression system uniquely combines relatively high protein yields with the intrinsic cellular capacity to perform complex post-translational modifications, such as N-linked glycosylation, disulfide bond formation, and phosphorylation, which are required for proper protein folding and biological activity [73,74]. These characteristics have made insect-cell expression systems useful for producing transporters, receptors, ion channels, and other membrane-associated proteins that are difficult to express in bacterial systems. This is particularly relevant to the targets considered in this review because AQP1, SGTP4, and parasite ABC transporters require membrane insertion and preservation of multi-pass transmembrane architecture, whereas GP63 and Sm-TSP-2 contain extracellular regions whose folding may depend on eukaryotic processing. Insect cells can therefore provide a useful compromise between expression capacity and eukaryotic processing for constructs that are poorly behaved in bacterial or yeast systems [73,74].

Another important advantage of BEVS is its compatibility with contemporary membrane-protein stabilization methods. Recombinant proteins produced in insect cells can readily be incorporated into nanodiscs, amphipols, styrene–maleic acid lipid particles (SMALPs), or proteoliposomes before cryo-EM imaging [75,76,77]. Preserving the lipid environment during purification reduces conformational instability and improves the likelihood of obtaining biologically relevant structures. The combination of insect-cell expression and membrane-mimetic technologies has therefore become a standard workflow for many challenging membrane proteins investigated by single-particle cryo-EM [76,77]. For *Leishmania* and *Schistosoma* targets, the main advantage of the baculovirus–insect cell system is therefore its ability to produce membrane-inserted proteins at a scale compatible with downstream purification and structural screening. AQP1, SGTP4, and ABC transporters could benefit from this platform when proper membrane insertion is limiting, whereas GP63 and Sm-TSP-2 may require additional assessment of glycosylation and extracellular domain integrity. In particular, the insect-cell product should not be automatically regarded as equivalent to the native parasite protein, as parasite-specific glycan structures, lipid composition, and membrane-associated interactions may not be reproduced. These differences should be evaluated experimentally before interpreting a recombinant structure as representative of the native target.

### 3.4. Mammalian Expression Systems

Mammalian expression systems provide a more native-like eukaryotic environment for proteins requiring complex folding, post-translational modification, membrane trafficking, or oligomerization [76,77,78,79]. Their main relevance to the parasite targets considered here lies in situations where simpler expression systems fail to preserve the structural features required for functional analysis. This may be particularly important for glycosylated or surface-associated targets such as GP63 and Sm-TSP-2, for which extracellular folding and post-translational processing may influence epitope presentation and structural stability. Mammalian systems may also be considered for AQP1, SGTP4, or parasite ABC transporters when membrane insertion or conformational stability remains problematic in insect cells. The principal disadvantages are the higher cost, slower production, and generally greater experimental complexity compared with bacterial, yeast, and insect-cell systems [77,78,79]. Mammalian expression should therefore be used selectively rather than assumed to be the preferred system for all parasite targets. For the proteins considered in this review, their value would be greatest when a more native-like eukaryotic environment is experimentally shown to improve folding, stability, or functional activity sufficiently to justify the additional production burden.

High-resolution structural determination requires homogeneous protein preparations that retain their native conformation throughout purification and vitrification [80]. Mammalian expression can, for some targets, improve the preservation of folding, post-translational processing, membrane trafficking, or oligomeric states relevant to structural analysis, thereby increasing the likelihood of obtaining high-quality cryo-EM reconstructions [80]. Optimization of transfection protocols, expression vectors, and culture conditions is therefore frequently required to obtain sufficient quantities of recombinant protein for cryo-EM analysis [78,79]. For the proteins considered here, mammalian expression is most useful when a more native-like eukaryotic environment is experimentally shown to improve folding, stability, post-translational processing, or functional activity sufficiently to justify the additional production burden.

### 3.5. Cell-Free Expression Systems

Cell-free protein synthesis (CFPS) provides an alternative route for producing recombinant proteins without relying on living host cells [81,82]. Its open reaction environment allows expression conditions, membrane mimetics, labels, and reaction components to be adjusted rapidly, making it useful for screening constructs that are toxic, unstable, or poorly expressed in conventional hosts. Integration with liposomes, nanodiscs, bicelles, or related systems can also support membrane insertion during synthesis, which is relevant to difficult multi-pass targets [81,82,83]. For the targets considered here, CFPS may be most valuable as a rapid screening platform rather than as the default production system. AQP1, SGTP4, and parasite ABC transporters could be tested in parallel with different membrane mimetics to determine whether membrane insertion and stability can be achieved before committing to larger-scale cell-based expression. The approach may also be useful for testing truncated constructs or engineered variants of difficult surface proteins. However, the absence of the full cellular processing environment remains a limitation for proteins whose structural properties depend strongly on glycosylation, membrane trafficking, or multiprotein assembly [81,82,83]. Thus, CFPS is best used as a complementary platform for identifying workable constructs and membrane environments rather than as a replacement for eukaryotic expression systems.

**Table 3:** Expression Strategies for Structural Characterization of *Leishmania* and *Schistosoma* Targets by Cryo-EM.

Expression System	Main Advantages	Target-Specific Structural Bottleneck	Most Relevant Targets in This Review	Likely Role in Structural Workflow
**Bacterial** **(*E. coli*)**	Rapid, inexpensive, scalable expression [69,70]	Poor parasite-like PTMs and difficulty maintaining correctly folded multi-pass membrane proteins	Soluble domains/fragments of GP63 and Sm-TSP-2; selected mutants or domains of AQP1, SGTP4 and ABC transporters	Construct screening, biochemical studies and domain production rather than intact membrane-protein preparation
**Yeast**	Eukaryotic folding, secretion and scalable production [71,72]	Non-native glycosylation and membrane composition may affect folding or stability	Selected GP63/Sm-TSP-2 constructs; preliminary production of parasite membrane proteins	Intermediate expression platform when eukaryotic processing is required
**Baculovirus-insect cells**	High expression capacity with eukaryotic processing and membrane insertion [73,74,75,76,77]	Parasite-specific glycosylation and lipid composition are not fully reproduced	AQP1, SGTP4, ABC transporters, GP63 and Sm-TSP-2	Strong candidate for intact membrane-protein production and downstream cryo-EM screening
**Mammalian cells**	More extensive eukaryotic processing and membrane trafficking [76,77,78,79,80]	High cost, slower production, and possible low yield	GP63 and Sm-TSP-2; difficult AQP1, SGTP4 or ABC transporter constructs requiring native-like processing	Selective option when insect-cell expression does not preserve structural or functional properties
**Cell-free systems**	Rapid construct testing and direct integration with membrane mimetics [81,82,83]	Folding, membrane insertion and complex PTMs may remain limiting	AQP1, SGTP4 and ABC transporters; selected engineered or truncated constructs	Rapid screening of constructs and membrane environments before scale-up

Expression-system selection should therefore be matched to the structural bottleneck of each target rather than applied uniformly to both parasites. For AQP1, SGTP4, and ABC transporters, efficient membrane insertion and preservation of transporter conformational states are central requirements. For GP63 and Sm-TSP-2, extracellular folding, glycosylation, membrane anchoring, and oligomeric or protein–protein interactions may be equally important. These requirements are further complicated by the *Leishmania* surface glycocalyx and the LPG-rich layer [43], as well as by the specialized Schistosoma tegument [44], none of which is fully recapitulated by conventional recombinant expression systems. Recombinant expression should therefore be regarded as a strategy for generating structurally informative material, with the extent to which that material represents the native parasite protein requiring experimental assessment.

### 3.6. Membrane-Mimetic Systems for Structural Stabilization

The preservation of native protein structure after extraction from host or parasite lipid bilayers remains a major challenge, as such extraction can destabilize target proteins, induce non-physiological conformational changes, and disrupt biologically relevant macromolecular complexes. Several membrane-mimetic systems have been developed to reconstitute the native lipid membrane bilayer during purification and cryo-EM analysis [77,84,85,86,87]. For the targets considered here, the choice of membrane mimic is particularly important because their structural bottlenecks differ. AQP1, SGTP4, and ABC transporters require a stable bilayer environment to preserve transmembrane packing and conformational states, whereas GP63 and Sm-TSP-2 may additionally depend on interactions involving their extracellular regions and membrane-anchoring elements. For parasite targets, these systems should therefore be selected according to the structural question being addressed. Nanodiscs may provide a defined bilayer for single-particle cryo-EM of purified AQP1, SGTP4, or ABC transporters, whereas lipid-retaining systems such as SMALPs or proteoliposomes may be useful when native lipid interactions are suspected to influence stability or function [84,86,87]. For GP63 and Sm-TSP-2, however, successful reconstitution would not by itself reproduce the complete parasite surface environment. Membrane-mimetic systems can preserve selected structural features, but their ability to reproduce the native parasite membrane and associated glycocalyx or tegument must be considered when interpreting resulting structures [43,44,75].

A practical solution to these limitations is to treat membrane-protein purification as a target-specific optimization process rather than as a single standardized procedure. A typical workflow begins with selection of an expression construct that preserves the required transmembrane and extracellular regions, followed by screening of expression conditions to identify a host and construct that provide sufficient protein while maintaining membrane insertion and stability. Membrane extraction can then be optimized by testing detergents with different chemical properties and concentrations, followed by affinity purification and size-exclusion chromatography to remove aggregates and isolate a homogeneous protein population. Detergents such as DDM, LMNG, digitonin, or GDN are commonly evaluated for membrane proteins because their effects on solubilization, stability, aggregation, and cryo-EM background can differ substantially between targets [84,88,89,90]. If detergent-solubilized protein remains unstable or loses function, exchange into amphipols or reconstitution into lipid nanodiscs can provide a more supportive membrane-like environment. Nanodiscs can also improve particle size and provide additional features that may facilitate particle alignment, whereas styrene–maleic acid lipid particles can preserve a portion of the native lipid environment by extracting membrane proteins directly with surrounding lipids [84,85,86,87,91]. For small targets such as AQP1, formation of complexes with high-affinity fiducial binders, including Fab fragments or nanobodies, provides another possible approach for increasing the effective particle mass and improving particle alignment [65,66,67]. These approaches should be evaluated sequentially using biochemical quality-control measures such as monodispersity, oligomeric state, thermal stability, ligand binding, or functional activity before proceeding to cryo-EM grid preparation. For AQP1, SGTP4, and parasite ABC transporters, this workflow could help identify conditions that preserve membrane insertion and conformational stability, whereas GP63 and Sm-TSP-2 may require additional optimization of glycosylation, extracellular-domain integrity, and membrane association. Importantly, these approaches are established methodological solutions for membrane-protein structural studies in other systems and should therefore be regarded as experimental options for the parasite targets discussed here rather than as already validated purification protocols for *Leishmania* or *Schistosoma* proteins.

### 3.7. Cryo-ET and In Situ Structural Biology

Single-particle cryo-EM enables structural analysis of isolated and purified macromolecules but generally removes them from the organized cellular environment in which many biological processes occur. Cryo-ET complements this approach by generating three-dimensional views of molecular assemblies within near-native cellular morphology [92,93,94]. Cryo-ET uniquely investigates membrane topography, host–parasite interfaces, tegumental structure, and endogenous intracellular complexes. The preservation of the spatial coordinates between proteins and their surrounding subcellular structures makes cryo-ET an imaging modality with capabilities that extend beyond conventional *in vitro* structural determination [92,93,94]. The application of cryo-ET to neglected tropical disease studies remains relatively limited. For *Leishmania* and *Schistosoma*, the main prospective value of cryo-ET lies in determining how parasite surface architecture, membrane-associated complexes, and host–parasite interfaces are organized in their native cellular context. Such information could eventually help define structural features relevant to target accessibility and delivery, but the direct translation of cryo-ET observations into effective nanomedicines has yet to be experimentally demonstrated.

Cryo-EM and cryo-ET can also provide complementary information on nanoparticle–membrane interactions, including membrane deformation, particle attachment, uptake, and intracellular trafficking [95,96]. For parasitic infections, however, evidence from non-parasitic membrane systems should be treated as a mechanistic precedent rather than as direct evidence of nanoparticle behaviour at the *Leishmania* or *Schistosoma* surface. Future studies combining parasite-derived membranes, purified target proteins, or intact parasite preparations with cryo-ET could help determine whether structural features identified in isolated systems are retained under more biologically relevant conditions.

## 4. Translating Cryo-EM Structures into Nanomedicine

High-resolution cryo-EM structures provide molecular information that can support the translation of protein structures into targeted therapeutic approaches [24,27]. In neglected tropical diseases, such structural information could contribute to antiparasitic drug discovery, which remains constrained by limited target selectivity, toxicity, treatment resistance, and challenges in achieving adequate drug concentrations at the site of infection [21,22,23,32]. Nanomedicine may provide a means of translating experimentally supported structural information into more targeted therapeutic methods, provided that target accessibility, ligand specificity, and therapeutic efficacy are subsequently established. In this review, “precision” refers to the use of molecular and structural information to improve selectivity for a defined biological target, whereas “targeted” nanomedicine refers to a nanocarrier engineered to recognize that target through a defined ligand–target interaction. “Parasite-targeted nanomedicine” is used more specifically to refer to systems in which the therapeutic carrier interacts with a parasite-associated molecular target rather than merely promoting uptake by host cells. This distinction is particularly important for *Leishmania*, where macrophage-directed uptake can improve delivery to infected cells but does not by itself demonstrate recognition of the intracellular parasite. Evidence for parasite-specific targeting should therefore include experimental confirmation of target accessibility in the native parasite, ligand–target binding or selective association, appropriate controls demonstrating reduced or absent interaction when the target is unavailable or blocked, and a biological effect that is linked to target engagement. Increased nanoparticle accumulation in infected macrophages alone should not be interpreted as evidence of parasite-specific targeting.

Surface localization alone, however, does not establish that a parasite protein is accessible to a nanocarrier in its native biological structure. In *Leishmania*, the LPG-rich glycocalyx forms a dense surface layer that can influence the exposure and interactions of available surface-associated molecules [43,97]. In *Schistosoma*, the specialized syncytial tegument and its associated membranocalyx constitute the principal host-facing surface and contain numerous membrane-associated proteins, transporters, glycans, and lipids [97]. Consequently, the accessibility of an individual target may depend on its orientation, abundance, membrane organization, glycosylation, and association with other surface components (Table 4). These features may also complicate structural characterization, as recombinant preparations may not reproduce the organization of the native parasite surface. Structural interpretation should therefore consider the native surface environment when assessing whether a candidate epitope or binding site is realistically accessible to a nanocarrier. The delivery route also differs substantially between the two parasite systems. *Leishmania* amastigotes reside predominantly within host macrophages, creating a two-stage targeting problem in which a nanocarrier must first reach infected host cells and subsequently deliver its payload in a manner that permits effective interaction with the intracellular parasite. Nanoparticle-based macrophage targeting has already been investigated as a strategy for improving anti-leishmanial drug delivery, including mannose-functionalized and other macrophage-directed systems [98]. In schistosomiasis, many candidate targets are associated with the host-exposed tegument of the extracellular worm, potentially providing a more direct interface for ligand-mediated recognition. Primary studies have now investigated antibody-functionalized nanocarriers against *Schistosoma mansoni*, including anti-calpain antibody-functionalized nanoliposomes and antibody-conjugated nanomicelles. These studies provide experimental evidence that antibody-functionalized nanocarriers can be evaluated against schistosome infection, but they do not establish that the parasite structural information discussed in this review has already been used to design or validate a structure-guided ligand–target interaction [19,99,100]. These biological differences should be considered when translating structural information into nanocarrier design.

### 4.1. Structure-Guided Targeted Drug Delivery

**Figure 3:**
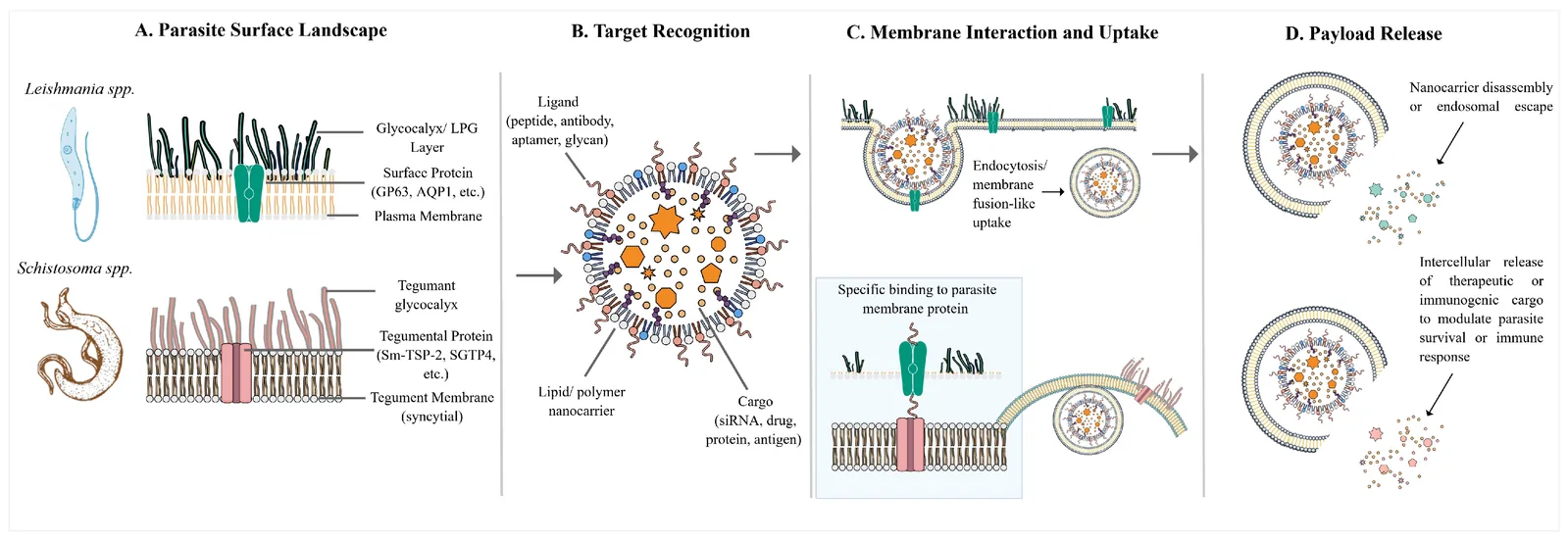
Parasite-Targeted Nanocarrier: Structural Recognition and Intracellular Delivery (Parasite-Specific Concept) (created with Mind the Graph software: https://mindthegraph.com). Schematic representation of a prospective structure-guided nanotherapeutic strategy in which a functionalized nanocarrier recognizes an accessible parasite-associated membrane protein and delivers its therapeutic payload. The illustration emphasizes target recognition, membrane interaction, cellular uptake, and intracellular payload delivery. The illustration represents a proposed mechanism rather than an experimentally established cryo-EM-guided delivery system for the parasite targets discussed in this review.

Structural information can inform nanocarrier design by identifying molecular features that are accessible and functionally relevant on the parasite. For example, an experimentally characterized surface-exposed domain or ligand-binding pocket could guide the selection or optimization of an antibody, peptide, or other targeting ligand for nanoparticle functionalization [62,101]. However, such structure-guided ligand selection remains prospective for the parasite targets considered here because direct validation of ligand binding to these targets in their native parasite context is limited. For *Leishmania*, primary nanomedicine studies have demonstrated targeted delivery against the infection, including macrophage-directed mannosylated chitosan nanoparticles, but these studies do not establish direct binding of the targeting ligand to a parasite membrane protein [98]. For parasite-specific targets such as AQP1 or SGTP4, structural information on extracellular loops and the orientation of exposed regions could help formulate testable hypotheses about ligand accessibility, but target-specific binding and therapeutic benefit would still require experimental validation (Figure 3).

### 4.2. Lipid Nanoparticles and Liposomal Delivery Systems

Lipid-based nanocarriers are important drug-delivery platforms owing to their formulation flexibility, biocompatibility, and ability to encapsulate a range of therapeutic agents [102,103]. In leishmaniasis, lipid-based formulations have been investigated extensively, and liposomal amphotericin B represents an established clinical application. Clinical studies have demonstrated high efficacy and lower toxicity of liposomal amphotericin B compared with conventional amphotericin B formulations in visceral leishmaniasis [104]. This example demonstrates how formulation can alter the therapeutic profile of an established antiparasitic drug, although its development was not based on a cryo-EM structure of a parasite target. Additional liposomal formulations of amphotericin B continue to be investigated to improve efficacy, administration, and tolerability [102]. Cryo-EM and cryo-ET can also provide structural information about lipid-based delivery systems, including particle morphology, size, internal organization, and lipid arrangement, thereby supporting formulation characterization. Such information may contribute to formulation optimization, but improved targeting precision and therapeutic efficacy require subsequent biological validation.

The applications outlined in Table 5 show potential uses of structural information rather than established outcomes of cryo-EM-based parasite studies. The identification of a structural feature does not by itself establish target accessibility, ligand specificity, biological activity, or therapeutic efficacy; these parameters require independent experimental validation.

### 4.3. Nanovaccines and Structure-Guided Antigen Design

The identification and validation of protective antigens remain important challenges in vaccine development against neglected tropical diseases, including leishmaniasis and schistosomiasis [30,31,105]. For leishmaniasis, numerous parasite antigens have been investigated, but no vaccine is currently licensed for human use, and only a limited number of candidates have progressed toward clinical evaluation [30,105]. In schistosomiasis, several tegument-associated proteins have similarly been investigated as vaccine candidates. Sm-TSP-2 is a notable example because it is exposed at the parasite surface and has been investigated as a vaccine antigen. Importantly, the available structural evidence for Sm-TSP-2 is limited to its extracellular EC2 domain, which was characterized by solution NMR and deposited in the PDB as 2M7Z; the intact four-pass membrane protein has not been structurally resolved by cryo-EM [29,49].

Structural information can nevertheless contribute to rational antigen design by defining the three-dimensional organization of antigenic regions, identifying surface-exposed residues, and distinguishing conformational features that may influence antibody recognition [62,105]. Cryo-EM is particularly valuable when sufficiently large and stable antigen–antibody or antigen–nanoparticle assemblies can be obtained, because it can provide structural information on conformational epitopes and macromolecular complexes.

Nanoparticle-based vaccine systems may provide additional advantages by improving antigen stability, controlling antigen presentation, facilitating uptake by antigen-presenting cells, and enhancing immune responses [105,108]. Nanoparticle platforms can also be engineered to display antigens in repetitive or defined orientations, potentially increasing antigen recognition and B-cell activation [105].

Drug delivery and vaccine development require different forms of structural evidence. For therapeutic nanocarrier delivery, a parasite-associated target must be accessible to the carrier, selectively recognized by the targeting ligand, and linked to a measurable antiparasitic effect. In contrast, a surface-exposed epitope may be valuable for vaccine development because of its ability to induce antibody or cellular immune responses, but this does not by itself establish that the same epitope is suitable for nanoparticle-mediated drug delivery. For parasite vaccines, structural information is therefore primarily useful for refining antigen and epitope selection, antigenic conformation, and presentation, followed by experimental confirmation of immunogenicity and protection in relevant models. This distinction is important for *Leishmania* and *Schistosoma*, where structural accessibility for immune recognition should not be equated with targetability for therapeutic nanocarriers.

### 4.4. Barriers to Translation

Despite advances in cryo-EM and nanomedicine [19,20,24,27], translating parasite structural information into targeted nanotherapeutics remains limited by the scarcity of experimentally resolved structures for many biologically important *Leishmania* and *Schistosoma* membrane proteins. The remaining structural gap is linked to target-specific requirements for expression, purification, membrane stabilization, and preservation of biologically relevant conformations. These limitations are particularly important for parasite surface proteins whose recombinant preparations may not reproduce the organization, post-translational modifications, lipid interactions, or accessibility of the native parasite membrane.

A second challenge is the biological complexity of nanocarrier delivery. For intracellular *Leishmania* amastigotes, an effective formulation must first reach infected macrophages and subsequently deliver its payload to a location where the parasite can be exposed to a therapeutically effective concentration. For *Schistosoma*, surface-directed methods may offer a more direct route to tegument-associated targets, but successful targeting still depends on accessibility, ligand affinity, carrier stability, and retention at the parasite surface. These differences mean that a single nanocarrier strategy is unlikely to be equally applicable to both parasite systems.

Translation is also constrained by nanoparticle formulation stability, reproducibility, scalability, manufacturing requirements, biodistribution, toxicity, and regulatory considerations [109]. Importantly, promising activity in cell culture or experimental animal models does not establish clinical effectiveness. A realistic structure-to-nanomedicine pipeline should therefore proceed sequentially: experimentally supported target determination; structural identification of candidate pockets or epitopes; computational prioritization where appropriate; experimental confirmation of target accessibility and ligand binding; nanocarrier functionalization and physicochemical characterization; *in vitro* assessment of targeting and antiparasitic activity; and finally, *in vivo* evaluation of efficacy, biodistribution, pharmacokinetics, and safety.

## 5. Integration of Cryo-EM, Artificial Intelligence, and Computational Structural Biology

### 5.1. Target-Specific Application of Artificial Intelligence-Assisted Structural Prediction

Recent advances in AI-based protein structure prediction have enabled the generation of structural models for experimentally unresolved proteins [110,111]. In the present context, however, the value of these models depends on the structural evidence already available for each parasite target. For AQP1, where a full-length *L. donovani* structure remains experimentally unresolved, predicted models can provide a working representation of the four-pass membrane architecture and help identify residues or pore regions that may warrant experimental investigation. Such models may also provide starting structures for molecular dynamics simulations to examine membrane stability, pore dynamics, or ligand interactions, but predicted conformations should not be interpreted as experimentally established states.

A similar strategy may be useful for SGTP4, for which functional and localization evidence exists, but a high-resolution parasite-specific structure has not been established. AI-based prediction can provide hypotheses concerning its membrane topology, extracellular and intracellular regions, and possible substrate- or inhibitor-interaction sites. These predictions could help prioritize constructs, stabilizing mutations, or experimental conditions for subsequent structural studies. Their usefulness, however, remains dependent on model confidence and experimental confirmation.

The situation differs for GP63 and Sm-TSP-2, for which experimental structural information is already available for defined regions. For GP63, the X-ray structure of the catalytic domain provides an experimentally supported reference for examining unresolved regions, whereas AI-based modelling can be used to examine unresolved regions or to generate hypotheses about the relationship between the catalytic domain and the membrane-associated protein. For Sm-TSP-2, the experimentally determined EC2-domain structure provides information on an exposed region of the protein, while computational prediction may help investigate the unresolved full-length four-pass membrane architecture. In both cases, computational models should be regarded as extensions of the available experimental evidence rather than replacements for it.

AI can also contribute at the stage of cryo-EM data interpretation [112]. Neural-network approaches developed for heterogeneous cryo-EM and cryo-ET datasets can assist the analysis and reconstruction of challenging density data. This may become particularly relevant if parasite membrane proteins or tegument-associated complexes are eventually obtained in multiple conformational states. In such cases, AI-assisted analysis could help distinguish structural heterogeneity and improve the interpretation of experimental density. It would not, however, overcome fundamental limitations arising from poor particle quality, inadequate sample preparation, low abundance, or inappropriate membrane environments. Overall, AI-based prediction is most appropriate here to bridge specific gaps among sequence information, preliminary structural hypotheses, and experimental structural determination. It can help determine which regions, constructs, conformations, or interactions warrant further investigation, but it cannot, by itself, establish the native structure, membrane organization, ligand accessibility, or therapeutic relevance of AQP1, GP63, Sm-TSP-2, or SGTP4. Therefore, experimental structural and functional validation remains essential (Tabel 6).

### 5.2. Molecular Docking, Molecular Dynamics Simulation, and Structure-Based Drug Discovery

For the targets considered in this review, molecular docking is most useful when an experimentally determined structure or a well-supported structural model is available to define a plausible binding region. For GP63, the experimentally determined catalytic-domain structure provides a direct structural model for investigating candidate inhibitor interactions [28]. For unresolved membrane proteins such as AQP1 and SGTP4, docking would instead depend on computationally predicted structures and should therefore be interpreted as hypothesis generation rather than direct structural evidence. Similarly, modelling of the unresolved regions of Sm-TSP-2 could provide a preliminary model for examining potential interactions, but experimentally accessible sites would still require validation. Docking can assist compound prioritization against these targets [113], but predicted poses and scores do not establish binding or biological activity. Their reliability depends on receptor quality, ligand preparation, scoring functions, conformational sampling, and treatment of protein flexibility (Tabel 6).

Molecular dynamics (MD) simulations provide a complementary approach for examining the conformational behaviour of parasite proteins and their complexes over time [106,107]. Their application should likewise depend on the structural status of each target. For AQP1 and SGTP4, MD simulations of predicted membrane-protein models could be used to examine structural stability, pore or transporter dynamics, and interactions with membrane lipids, thereby identifying features that could subsequently be tested experimentally. For GP63, simulations can be applied to the experimentally determined catalytic domain to investigate ligand stability and local conformational changes, while simulations of Sm-TSP-2 could help examine the behaviour of its experimentally characterized EC2 domain or computational models of unresolved regions. Membrane-specific simulations may be particularly informative for AQP1, SGTP4, and parasite ABC transporters, as their structural behaviour depends on the surrounding lipid environment (Table 6). Nevertheless, MD trajectories remain dependent on the starting model, force field, simulation conditions, and sampling time and should therefore be interpreted as mechanistic hypotheses rather than experimental observations [106,107].

For membrane-associated targets, the choice of bilayer composition should also reflect the biological membrane being modelled rather than relying on a generic phosphatidylcholine bilayer. The surface membrane of *Leishmania donovani* contains phosphatidylcholine, phosphatidylethanolamine, phosphatidylinositol, phosphatidylserine, glycolipids, and substantial neutral lipid and sterol fractions [114]. The tegumental membranes of *Schistosoma mansoni* are likewise compositionally distinct from host membranes and contain phosphatidylcholine, phosphatidylethanolamine, phosphatidylserine, and phosphatidylinositol species, together with parasite-enriched lysophospholipids and unusual fatty-acid-containing phospholipids [115,116]. These differences are relevant when modelling AQP1, SGTP4, or parasite ABC transporters because lipid composition can influence membrane thickness, protein–lipid interactions, conformational stability, and access of ligands or substrates to membrane-embedded regions. All-atom MD can provide detailed information on residue-level interactions, lipid contacts, hydration, and local conformational changes, but is generally limited in accessible spatial and temporal scales. Coarse-grained MD reduces this computational cost and can therefore be useful for examining larger membrane assemblies, lipid redistribution, protein–lipid interactions, and longer-timescale conformational behaviour [117,118]. For parasite targets, coarse-grained simulations can first examine how candidate membrane environments influence protein organization, followed by atomistic simulations of selected regions or states where residue-level mechanisms require investigation. The membrane composition used in either approach should therefore be justified from available parasite lipid data and treated as an important model parameter rather than a generic simulation condition.

**Table 6:** Computational Approaches Complementing Cryo-EM in Structural Parasitology.

Computational Approach	Primary Purpose	Cryo-EM Integration	Application in Drug/Nanotherapeutic Development	Outputs	Parasite-Specific Application
**AI-based structural prediction (e.g., AlphaFold, RoseTTAFold)** [110,111,119,120]	Predict three-dimensional protein structures from amino acid sequences where experimental data are unavailable	Provides structural hypotheses for unresolved targets and may complement experimentally resolved cryo-EM structures by modelling unresolved regions or alternative conformations	Target prioritization, structural annotation, candidate pocket and epitope identification	Predicted structural models, domain organization, confidence scores	AQP1 and SGTP4: generate preliminary full-length membrane-protein models where experimental structures are unavailable; Sm-TSP-2: complement the experimentally resolved EC2 domain by modelling unresolved regions; GP63: assist analysis of unresolved regions surrounding the experimentally characterized catalytic domain. Predictions require experimental validation
**Molecular Docking** [113]	Predict ligand–protein binding modes and estimate binding affinity	Uses experimentally determined or computationally predicted structures as receptor models for ligand screening	Structure-guided identification and prioritization of candidate inhibitors and targeting ligands	Predicted binding poses, docking scores, interaction profiles	GP63: supports investigation of ligand interactions using an experimentally determined catalytic-domain structure; AQP1 and SGTP4: can prioritize compounds against predicted binding regions; Sm-TSP-2: may assist exploratory analysis of candidate surface interactions. Results obtained from predicted structures remain hypotheses requiring experimental validation
**Molecular Dynamics Simulations** [106,107]	Investigate protein flexibility, conformational stability, and molecular interactions over time.	Can evaluate structural stability and conformational behaviour of experimentally determined cryo-EM structures or computationally predicted protein models and their associated protein–ligand complexes	Evaluation and optimization of candidate ligands and investigation of structural features that may influence target recognition	RMSD, RMSF, hydrogen-bonding patterns, conformational transitions, free-energy evidence	AQP1 and SGTP4: assess stability and conformational behaviour of predicted membrane-protein models; GP63: examine ligand interactions with the experimentally characterized catalytic domain; Sm-TSP-2: investigate domain or modelled full-length dynamics. Membrane proteins should be simulated in appropriate lipid environments where required.
**Virtual Screening** [121,122]	Evaluate large chemical libraries against selected therapeutic targets	Uses experimentally resolved or computationally predicted structures as templates for computational screening	Identification and prioritization of potential antiparasitic compounds and drug-repurposing candidates	Ranked hit compounds, predicted binding scores and interaction profiles	Can support prioritization of compounds against structurally characterized or modelled *Leishmania* and *Schistosoma* targets; predicted activity requires experimental confirmation
**MM/GBSA and binding free-energy calculations**	Estimate relative binding free energies after docking or MD simulations	Can refine and rank interactions identified through docking and MD using experimentally determined or modelled structures	Lead prioritization and comparative evaluation of ligand binding	ΔG_bind_, energetic components, residue energy decomposition	May help prioritize ligands targeting parasite proteins, including exposed or tegument-associated targets; calculated affinities should not be interpreted as experimental measurements
**Density Functional Theory (DFT)**	Investigate electronic structures, molecular reactivity, and interaction properties	Provides atomistic and electronic-level analysis of ligand or functional groups identified through structural and docking studies	Ligand optimization, analysis of reactive groups and evaluation of chemical properties relevant to functionalization	HOMO–LUMO energies, molecular electrostatic potential, chemical reactivity descriptors	May support mechanistic analysis and optimization of compounds or targeting ligands directed toward parasite-associated targets; its contribution is complementary rather than a substitute for structural or biological validation
**Protein–protein interaction (PPI) modelling**	Characterize interfaces between parasite proteins or between parasite and host proteins	Combines structural information with experimentally identified or predicted interaction interfaces	Identification of potential therapeutic targets, vaccine candidates and host–parasite interaction sites	Interface residues, interaction models, binding hotspots and interaction networks	Can help investigate host–parasite interfaces in *Leishmania* and *Schistosoma* and identify candidate regions for further experimental validation
**Machine learning-assisted drug discovery** [123,124]	Identify patterns linking molecular structure to biological activity and predict candidate compounds	Can integrate structural information with biochemical, pharmacological, and screening datasets associated with experimentally characterized targets	Compound prioritization, activity prediction and, where appropriate, ADMET profiling	Predictive models, activity scores, classification, or regression outputs	Can integrate predicted or experimentally supported structural features with biochemical or screening data to prioritize compounds against AQP1, GP63, SGTP4, or other validated parasite targets. Its usefulness will depend on the availability and quality of parasite-specific training data

RMSD = root-mean-square-deviation, RMSF = root-mean-square-fluctuation, HOMO = highest occupied molecular orbital, LUMO = lowest unoccupied molecular orbital, MM/GBSA = molecular mechanics/generalized Born surface area, ADMET = absorption, distribution, metabolism, excretion, and toxicity.

### 5.3. Computational Approaches in Nanotherapeutic Design

Computational approaches may also provide an intermediate step between structural characterization and nanotherapeutic design. For GP63 and Sm-TSP-2, experimentally determined structural regions can serve as a starting point for examining candidate ligand- or epitope-accessible surfaces, whereas predicted models of AQP1 and SGTP4 can be used to generate preliminary hypotheses about exposed regions or binding sites. Molecular docking and simulation can then examine ligand orientation, interaction stability, and, where appropriate, the influence of the membrane environment [106,107,113]. Such analyses may help prioritize targeting ligands or formulation approaches for experimental testing, but they do not establish that a predicted site is accessible on the intact parasite or that a nanocarrier directed against it will produce therapeutic benefit.

Nanotherapeutic approaches have already been investigated in both leishmaniasis and schistosomiasis, but these studies should not be interpreted as evidence that cryo-EM-derived parasite structures have already guided nanocarrier development. In *Leishmania*, ligand-functionalized nanoparticles have been investigated for targeted delivery of anti-leishmanial agents [112,113]. In *Schistosoma*, nanostructured formulations and targeted delivery approaches have been explored to improve the therapeutic activity and delivery of antiparasitic agents, including approaches directed toward tegument-associated molecular targets [19,64]. These studies demonstrate the feasibility and therapeutic interest of parasite-directed nanomedicine, but they have not established a general structure-guided workflow based on experimentally resolved cryo-EM structures of the corresponding parasite targets. Cryo-EM could therefore strengthen existing nanomedicine approaches by providing experimentally supported structural information for target selection and ligand design, but this application remains prospective for the parasite targets considered here.

### 5.4. Current Limitations of Computational Structural Approaches

Computational predictions remain dependent on the quality of the available structural models and their conformational states [123]. Static structures may not fully capture dynamic conformational changes, transient macromolecular interactions, or fluctuations in the biological environment [124]. Similarly, molecular docking and molecular simulations rely on inherent algorithmic assumptions and simplified force fields, such as fixed solvent states, simplified charge states, scoring functions, and rigid receptor backbones, which can skew predictive accuracy. The uncertainty associated with computational predictions increases when experimental structural or functional data are limited [125]. These approaches are therefore best regarded as complementary tools that guide experimental investigation rather than as replacements for experimental structural and biological validation. Future work should therefore focus on integrating computational predictions with experimentally resolved structures, parasite-specific biochemical data, and functional assays. For AQP1, SGTP4, and the unresolved regions of Sm-TSP-2 and GP63, this integrated approach could help determine which structural hypotheses are sufficiently well supported to warrant experimental testing and, subsequently, whether any identified features are relevant to therapeutic targeting.

## 6. Knowledge Gaps and Potential Research Opportunities

A major structural gap is the limited availability of native-context information for parasite surface proteins. Glycan heterogeneity, native lipid interactions, and complex surface organization may influence structural interpretation, particularly in the *glycocalyx of Leishmania and the tegument of Schistosoma*. Cryo-ET could provide complementary information on these native architectures, but its application to these parasites remains limited [72].

Another major knowledge gap concerns the connection between experimentally determined structures and nanotherapeutic design. Most antiparasitic nanoparticle systems have been developed primarily on the basis of pharmacological, physicochemical, or formulation considerations rather than on the direct use of experimentally resolved parasite structures [19,20,64]. Consequently, structure-guided identification of accessible epitopes, ligand-binding pockets, or protein–nanocarrier recognition sites remains largely prospective for the parasite targets considered in this review [19,20,64]. Surface localization should not be interpreted as evidence of nanocarrier accessibility. In *Leishmania*, the glycocalyx and LPG-rich surface layer may restrict access to available targets, whereas the syncytial tegument of *Schistosoma* presents a complex host-facing surface in which target exposure depends on protein abundance, orientation, membrane organization, and association with other tegumental components.

Vaccine development presents a further unresolved area. Several parasite antigens have demonstrated immunogenic potential, but the identification of protective epitopes and their accessibility within native parasite surfaces remains incomplete [30,31,105]. Experimentally determined structures could support the identification of exposed antigenic regions and inform antigen-display strategies, including nanoparticle-based vaccine platforms. However, structural accessibility alone does not establish protective immunity, and candidate epitopes require experimental assessment of immunogenicity and protection.

## 7. Future Perspectives

Future work should first focus on generating experimentally supported structures for parasite targets that currently lack complete structural characterization. For membrane proteins such as AQP1, SGTP4, and parasite ABC transporters, this will require optimizing expression, membrane stabilization, and sample preparation based on the properties of each target. Cryo-EM and cryo-ET may be particularly useful for determining conformational states, membrane-associated assemblies, and native parasite surface organization that cannot be adequately resolved from computational models alone.

Computational methods will continue to have a complementary role in prioritizing unresolved targets, identifying candidate binding regions, and interpreting experimental structures. Their use should remain closely linked to biochemical, structural, and functional validation. For nanomedicine, the next practical step is not simply to identify additional structural models, but to test whether structurally defined parasite features are accessible in the native parasite and can be recognized selectively by validated ligands. Subsequent studies should determine whether such ligand–target interactions can be retained after nanocarrier functionalization and whether they produce measurable improvements in parasite-associated drug delivery and antiparasitic activity.

The requirements will differ between the two parasite systems. In *Leishmania*, studies must account for the intracellular location of amastigotes and distinguish macrophage uptake from direct parasite targeting. In *Schistosoma*, surface-directed approaches can be evaluated against tegument-associated targets, but accessibility should be confirmed within the native tegumental environment. For vaccine applications, structural information should similarly be linked to experimental assessment of antigenicity, immunogenicity, and protection rather than assumed to predict vaccine efficacy.

## 8. Conclusions

Leishmaniasis and schistosomiasis remain important neglected tropical diseases for which improved therapeutic and vaccine approaches are needed. The parasite membrane-associated targets discussed in this review have diverse roles in transport, host–parasite interaction, immune modulation, and parasite survival, but their structural characterization remains limited by membrane dependence, conformational variability, expression difficulties, glycosylation, and the complexity of native parasite surfaces. Taken together, the available evidence supports a prospective role for structural information in parasite-directed therapeutic and vaccine development, but stronger experimental links between molecular structure, target accessibility, molecular interaction, and biological activity are required before such applications can be considered established.

## Figures and Tables

**Table 1:** Major Therapeutic Challenges in Leishmaniasis and Schistosomiasis and the Potential Contribution of Cryo-EM.

Challenges	Leishmaniasis	Schistosomiasis	Consequences for Disease Management	Potential Future Contribution of Cryo-EM (Prospective)	Supporting References
**Drug resistance**	Resistance to antimonials is well documented, and reduced susceptibility to miltefosine has been reported in some settings.	Reduced praziquantel susceptibility has been reported in some field isolates and endemic settings.	Reduced treatment effectiveness and risk of disease persistence	Identification of drug-binding pockets and resistance-associated structural changes	[21,22,23]
**Limited therapeutic options**	Few approved drugs are available	Heavy reliance on praziquantel	Vulnerability to treatment failure	Discovery of novel molecular targets for drug design and development	[24,25]
**Drug toxicity**	Amphotericin B and antimonials may cause serious adverse effects	Praziquantel is generally well tolerated, but repeated treatment may be required because of reinfection and reduced susceptibility	Reduced patient compliance and treatment limitations	Structure-guided development of selective therapeutics with reduced off-target effects	[26,27]
**Poor target characterization**	Many membrane proteins remain structurally unresolved	Numerous tegument-associated proteins remain poorly characterized	Slower therapeutic discovery	High-resolution visualization of therapeutic targets	[28,29]
**Vaccine limitations**	No licensed human vaccine	No licensed human vaccine	Continued dependence on chemotherapy	Structural mapping of protective epitopes for vaccine design	[30,31]
**Ineffective drug delivery**	Intracellular localization within host macrophages complicates access of drugs to intracellular amastigotes	The tegument constitutes a major host–parasite interface and can influence drug accessibility to parasite tissues.	Reduced therapeutic concentration at target sites	Identification of surface-accessible targets for nanoparticle-mediated delivery	[32]
**Host–parasite adaptation**	Host adaptation and immune evasion involve dynamic molecular processes that remain incompletely resolved	Complex immune evasion mechanisms persist	Difficulty achieving long-term disease control	Visualization of protein conformational states involved in host interaction	[33,34]

**Table 2:** Structural Evidence and Therapeutic Relevance of Selected *Leishmania* and *Schistosoma* Targets.

Parasites	Protein Target	Cellular/Parasite Location	Biological Function	Therapeutic Relevance	Structural Evidence	Experimental Method	PDB ID	Structural Status	Supporting References
***Leishmania donovani***	Aquaglyceroporin 1 (AQP1)	Parasite plasma membrane	Aquaglyceroporin is involved in water/solute transport and implicated in antimonial uptake and resistance	Drug resistance and antimonial transport	Functional and biochemical evidence is available, but no experimentally resolved full-length *L. donovani* AQP1 structure was identified; its small membrane-protein architecture presents a potential single-particle cryo-EM alignment challenge	Functional/biochemical studies and computational modelling; prospective single-particle cryo-EM using fiducial binders and a membrane-mimetic system	-	Computational/Structurally unresolved	[21,46,51,52]
***Leishmania major***	Glycoprotein 63 (GP63)/Leishmanolysin	Parasite surface/membrane-associated	Surface zinc-dependent metalloprotease involved in host–parasite interaction and immune modulation	Vaccine and drug target	An experimentally determined structure of solubilized *L. major* leishmanolysin is available from X-ray crystallography; however, it does not represent the intact membrane-associated protein in its native parasite membrane environment	X-ray crystallography of solubilized/catalytic region; cryo-EM/cryo-ET are prospective for intact membrane-associated GP63	1LML	Experimental structure of solubilized protein; non-cryo-EM	[28,47,53]
***Leishmania*** **spp.**	ATP-Binding Cassette (ABC) transporters	Parasite plasma membrane	ATP-dependent membrane transport and drug efflux	Multidrug resistance and therapeutic targeting	Parasite-specific high-resolution structures remain limited; structural interpretation relies substantially on homologous ABC transporter structures and computational approaches	Experimental studies of homologues; computational modelling	-	Parasite-specific structure unresolved/underexplored	[48,54]
***Schistosoma mansoni***	Tetraspanin-2 (Sm-TSP-2)	Tegument/plasma membrane	Tegument organization and membrane-associated protein interactions	Vaccine candidate and tegumental target	An experimentally determined solution-NMR structure is available for the 81-residue extracellular EC2 domain, whereas the intact four-pass Sm-TSP-2 membrane protein remains structurally unresolved	Solution NMR of isolated EC2 domain; prospective cryo-EM of intact protein or complexes	2M7Z	Partial experimental structure; non-cryo-EM	[29,49]
***Schistosoma mansoni***	Glucose Transport Protein 4 (SGTP4)	Tegument/surface-associated	Glucose uptake across the tegument	Metabolic/transport target	Functional and localization evidence exists, but no experimentally resolved high-resolution structure of SGTP4 has been identified; its membrane-spanning architecture and conformational states therefore remain unresolved	Functional studies; computational modelling	-	Computationally modelled; experimentally unresolved	[50,55,56,57]
***Schistosoma*** **spp.**	Tegument-associated proteins	Tegument/surface-associated	Host interaction, nutrient uptake, membrane maintenance, and immune evasion	Drug, vaccine, and surface-targeting opportunities	Structural evidence varies among individual proteins; many tegument-associated membrane proteins remain unresolved	Multiple experimental/computational approaches	-	Heterogeneous; target-dependent	[10,58]

**Table 4:** Parasite-specific Structural Considerations for Targeted Nanomedicine in Leishmaniasis and Schistosomiasis.

Parasites	Structural/Biological Feature	Effect on Target Accessibility	Implication for Nanocarrier Design	Current Evidence Level
***Leishmania*** **spp.**	Glycocalyx and lipophosphoglycan (LPG)-rich surface layer	May influence exposure and accessibility of surface-associated proteins and alter interactions with extracellular materials	Nanocarrier ligands should be evaluated for accessibility to the intended target rather than assuming that surface localization guarantees binding	Prospective [43,98]
***Leishmania*** **spp.**	Surface and membrane-associated proteins	Accessibility may vary with parasite developmental stage, membrane organization, and surrounding surface components	Structural information could generate testable hypotheses about accessible epitopes or binding sites for targeted delivery, but direct ligand binding, target accessibility, and biological activity would require experimental validation	Prospective; target-dependent [46]
***Leishmania*** **spp.**	Intracellular localization within host macrophages	Nanocarriers must reach infected cells before parasite-target engagement can occur	Delivery systems may require both host–cell uptake and subsequent parasite-target recognition	Established biological constraint; targeting strategy remains under development [61,96,97]
***Schistosoma*** **spp.**	Specialized syncytial tegument	Tegument forms the principal interface between parasite and host and can regulate exposure of surface molecules	Target accessibility should be assessed in the native tegument rather than inferred solely from isolated recombinant proteins	Prospective; primary targeting evidence exists, but structure-guided ligand–target validation remains limited [19,99,100]
***Schistosoma*** **spp.**	Tegument-associated membrane proteins and glycans	Surface organization may influence antibody, ligand, and nanoparticle interactions	Structural characterization could help identify accessible extracellular regions suitable for targeting	Prospective [10,57,58]
***Schistosoma*** **spp.**	Extracellular surface exposed to host environment	Surface targets are potentially accessible without requiring intracellular parasite delivery, but accessibility can vary among targets	Surface-directed nanocarriers may be considered when target exposure and binding specificity are experimentally demonstrated	Target-dependent [10,49,57,58]

**Table 5:** Potential Contributions of Cryo-EM to Nanotherapeutic Development for Neglected Tropical Diseases.

Cryo-EM Structural Feature	Biological Significance	Application in Nanotherapeutic Design	Current Challenges/Future Opportunities
**Ligand-binding pockets**	Reveal active, allosteric, or ligand-interacting sites involved in substrate recognition and inhibitor binding	May inform selection of targeting ligands, structure-guided inhibitor development, and ligand-functionalized nanocarriers [61,101]; direct structure-guided nanocarrier development remains prospective for the parasite targets reviewed here	Experimentally resolved parasite structures remain limited; predicted binding sites and ligand–target interactions require biochemical and cellular validation
**Surface accessible epitopes**	Identify exposed antigenic regions available for immune recognition	May support nanovaccine development, antigen-display platforms, and epitope-focused vaccine design	Protective capacity and accessibility of candidate epitopes must be experimentally established; long-term immune responses remain insufficiently characterized [30,31,105]
**Transport channels and membrane pores**	Reveal molecular pathways involved in nutrient uptake, metabolite exchange, and drug transport [50,55,56]	May inform development of transport-targeting ligands and delivery strategies designed to exploit or modulate parasite transport pathways	Functional characterization of many parasite transporters remains incomplete, and structural accessibility in the native parasite membrane requires validation
**Protein–lipid interaction interfaces**	Identify lipid-dependent structural stabilization, membrane association, and interactions with surrounding membrane components	May inform optimization of lipid nanoparticles, liposomes, nanodiscs, and other biomimetic delivery systems	Parasite membrane composition and lipid–protein interactions remain incompletely characterized, limiting rational biomimetic carrier design.
**Conformational dynamics**	Reveal structural transitions associated with activation, inhibition, ligand binding, or changes in protein function [106,107]	May support identification of conformation-dependent targeting strategies and structure-guided inhibitor development	Dynamic structural information for parasite membrane proteins remains limited; the functional relevance of observed conformational states requires experimental confirmation
**Host–parasite interaction interfaces**	Identify molecular contacts involved in adhesion, invasion, immune evasion, and host adaptation [47,53]	May inform targeted nanoparticle functionalization, receptor-directed delivery, and development of anti-adhesion strategies	High-resolution structures of native host–parasite complexes remain scarce; interaction specificity and biological relevance require experimental validation
**Protein oligomerization and macromolecular assemblies**	Reveal quaternary organization and higher-order assemblies associated with biological activity	May inform multivalent targeting strategies and approaches aimed at disrupting functionally important protein assemblies	Native oligomeric states may differ from recombinant preparations; cryo-ET and complementary structural approaches may provide additional information in native cellular contexts
**Transient protein conformations**	Provide information on short-lived structural states associated with biological function	May support identification of transient or cryptic binding sites for future structure-guided therapeutics	Time-resolved and dynamic cryo-EM approaches remain underused in parasitology, and experimental validation of transient states remains challenging

## Data Availability

All source data generated in this study are available from the corresponding author upon request.

## Abbreviations

The following abbreviations are used in this manuscript:

Cryo-EM	Cryogenic Electron Microscopy
Cryo-ET	Cryo-Electron Tomography
NMR	Nuclear Magnetic Resonance
NTDs	Neglected Tropical Diseases
AI	Artificial Intelligence
LPG	Lipophosphoglycan
SGTP4	*Schistosoma mansoni* Glucose Transporter Protein 4
Sm-TSP-2	*Schistosoma mansoni* Tetraspanin-2
ABC	ATP-Binding Cassette Transporters
CFPS	Cell-Free Protein Synthesis
BEVS	Baculovirus Expression Vector System
GP63	Glycoprotein 63
AQP1	Aquaglycoporin 1
TMD	Transmembrane Domains
MDR	Multi-Drug Resistance
LUMO	Lowest Unoccupied Molecular Orbital
HOMO	Highest Occupied Molecular Orbital
DFT	Density Functional Theory
MM/GBSA	Molecular Mechanics/Generalized Born Surface Area
PPI	Protein–Protein Interactions
RMSD	Root-Mean-Square-Deviation
RMSF	Root-Mean-Square-Fluctuation
ADMET	Absorption, Distribution, Metabolism, Excretion, and Toxicity
